# Host Distribution Does Not Limit the Range of the Tick *Ixodes ricinus* but Impacts the Circulation of Transmitted Pathogens

**DOI:** 10.3389/fcimb.2017.00405

**Published:** 2017-10-11

**Authors:** Agustín Estrada-Peña, José de la Fuente

**Affiliations:** ^1^Faculty of Veterinary Medicine, University of Zaragoza, Miguel Servet, Zaragoza, Spain; ^2^SaBio, Instituto de Investigación en Recursos Cinegéticos IREC-CSIC-UCLM-JCCM, Ciudad Real, Spain; ^3^Department of Veterinary Pathobiology, Center for Veterinary Health Sciences, Oklahoma State University, Stillwater, OK, United States

**Keywords:** tick-borne pathogens, communities, habitat overlap, functional redundancy

## Abstract

Ticks, pathogens, and vertebrates interact in a background of environmental features that regulate the densities of ticks and vertebrates, affecting their contact rates and thence the circulation of the pathogens. Regional scale studies are invaluable sources of information about the regulation of these interactions, but a large-scale analysis of the interaction of communities of ticks, hosts, and the environment has been never modeled. This study builds on network analysis, satellite-derived climate and vegetation, and environmental modeling, quantifying the interactions between the tick *Ixodes ricinus* and the transmitted bacteria of the complex *Borrelia burgdorferi* s.l. in the Western Palaearctic. We derived the rates of contact of the tick with 162 species of vertebrates recorded as hosts, and the relative importance of each vertebrate in the circulation of the pathogen. We compiled more than 11 millions of pairs of coordinates of the vertebrates, deriving distribution models of each species and the relative faunal composition in the target territory. The results of the modeling of the distribution of the tick and its hosts, weighted by their importance in the circulation of *Borrelia* captured the spatial patterns of interactions that allow the circulation of the pathogen. Results indicate that both *I. ricinus* and *B. burgdorferi* s.l. are supported in the Western Palaearctic by complex communities of vertebrates, which have large distribution ranges. This high functional redundancy results in the pervasiveness of *B. burgdorferi* s.l., which depends on the gradient of contributions of the large community of vertebrates, instead of relying on a few dominant vertebrates, which was the prevailing paradigm. Most prominent reservoirs of the pathogen are distributed in specific regions of the environmental niche. However, literally dozens of potential reservoirs can colonize many other environmental regions, marginally but efficiently contributing to the circulation of the pathogen. These results consistently point to the need of evaluating the beta-diversity of the community of vertebrates acting as reservoirs of the pathogen to better know the interactions with the vector. They also demonstrate why the pathogen is so resilient to perturbations in the composition of the reservoirs.

## Introduction

The classic procedure to evaluate the potential distribution ranges of ticks has been the capture of their abiotic ecological relationships, with a main attention on climate, using more or less coherent series of records of the focal species and different modeling strategies. Modeling approaches may include variables other than climate, such as categories of the vegetation (which are qualitative proxies for climate), topological features (like slope or altitude) and several indexes derived from the habitat fragmentation and its connectivity (Estrada-Peña, 2003a,b; Brownstein et al., 2005; Li et al., 2012). Other than some advances in producing consistent datasets of climate features and general reviews about the most common gaps in modeling procedures (Estrada-Peña et al., 2013, 2015a), the approach of environmental modeling for ticks and the transmitted pathogens has not experienced significant improvements. The use of habitat fragmentation as an index impacting the resilience of the metapopulation of ticks provided a framework to test the effects of the hosts habitat corridors, and thus its contribution for supporting foci of tick-borne pathogens (Estrada-Peña, 2003a,b). However, field data have proven to be difficult to obtain for verifying this hypothesis (Linard et al., 2007; Lambin et al., 2010; Li et al., 2014; Pérez et al., 2016). Detailed predictions of how individual host species densities will change with fragmentation are not yet possible, despite their importance in the ecology of tick-borne pathogens (Kikpatrick et al., 2017).

In Europe, research has been focused on the tick *Ixodes ricinus*, because of its central role in the transmission of pathogens affecting human health (Medlock et al., 2013). Interest exists to understand the factors shaping tick local densities or the factors regulating the foci of pathogens that the tick transmits. It has been shown that different factors influence the acquisition, maintenance and transmission of pathogens by ticks (de la Fuente et al., 2017). Initial studies demonstrated that the distribution of the tick could be adequately sketched from the main environmental variables in large territories, but also pointed out that the prediction of abundance at regional scales is complex (Vanwambeke et al., 2016). Biotic relationships between a species of tick and its hosts are rarely considered in evaluating the presence and/or abundance of the tick in a territory. Ticks with a wide range of hosts have generalist feeding habits and feed on a large number of vertebrate species. Therefore, it is implicitly assumed that if the environmental factors are suitable, the tick could colonize an area without further evaluation of the biotic component represented by the hosts. This has yet to be empirically proven for most generalist species of ticks, but it is known that *Hyalomma marginatum* has biotic restrictions for colonization in a relatively large area in southern France because of the low densities of large animals necessary to feed the adults (Vial et al., 2016).

Feeding rates of ticks on different vertebrates result from both the availability of the hosts, and their suitability as hosts for the tick. These diverse relationships between hosts and ticks have an impact on the circulation of tick-borne pathogens. Each species of vertebrate has a different capacity to support the circulation of some pathogens. This is well studied for the bacteria of the group *Borrelia burgdorferi* s.l., transmitted by ticks of the complex *Ixodes ricinus*, for which evidence indicates that interactions between the pathogen and reservoirs result in the circulation of combinations of pathogen species according to the availability of prevailing reservoirs for the tick (Kurtenbach et al., 2002; Margos et al., 2011). Studies on *Ixodes scapularis*, which is the vector of the pathogen in parts of USA, demonstrated that, even if several host species are available for feeding the ticks, the carrying capacity of each host is different, some of them feeding a large number of ticks, some other allowing small fractions of the tick population to feed on them (LoGiudice et al., 2003). Further studies demonstrated the differential ability of different host species to feed ticks (Eisen et al., 2004; Castro and Wright, 2007; Barbour et al., 2015). Studies on *I. ricinus* have focused on the durability of the enzootic cycles of *B. burgdorferi*, which depend on the density and abundance of the various vertebrate reservoirs (Hofmeester et al., 2016).

A complex pattern emerges, driven by the abundance of the tick and the faunal composition of vertebrates, serving as hosts for the tick and/or reservoirs for the pathogen, which are not necessarily the same species. Environmental variables shape the distribution of *I. ricinus* and its vertebrate hosts, while the later act as filters of the foci of tick-transmitted pathogens, supporting variable feeding success of the tick and allowing or blocking the circulation of the genospecies of *B. burgdorferi* s.l. These interactions result from (i) the presence/absence and abundance of different host species for the tick, (ii) the degree of spatial overlap between the host species and the tick, (iii) the seasonality of the partners (allowing the temporal overlap and contact for efficient transmission of the pathogen), and (iv) the reservoir capacity of the set of hosts available to feed the ticks. The geographical distribution of *B. burgdorferi* s.l. in questing ticks is therefore a function of the densities of different host species, their capacity to feed ticks and their ability to transmit the bacteria to those ticks.

A comprehensive review of the uncertainties regarding the dynamics of *B. burgdorferi* s.l. has been recently published (Kikpatrick et al., 2017). These authors explicitly stated the need of predicting the nymphal infection prevalence from vector-host-pathogen interactions which “requires data on the fraction of larval ticks that feed on each host species, the fraction of hosts of each species that are infected, and the reservoir competence of these hosts for transmitting *Borrelia* spirochetes” (Kikpatrick et al., 2017). Because the intrinsic difficulties to obtain these estimates from field studies and extrapolate to different sites and time periods, a holistic approach is necessary, connecting the parts in a common framework.

We previously produced a coherent set of records of the tick *I. ricinus* in its Western Palaearctic distribution area, and its recorded hosts (162 species, more than 11 millions of geo-referenced records) and demonstrated that the methodological approach termed network analysis is adequate to examine the biotic relationships between ticks, hosts, and pathogens (Estrada-Peña et al., 2015b). This study was aimed to examine how the environmental variables impact the availability of hosts for the tick in the Western Palaearctic shaping the circulation of the pathogens of the group *B. burgdorferi* s.l. The results provided the first large scale predictive assessment of the contribution of 162 species of vertebrates to the circulation of *B. burgdorferi* s.l. in the target territory identifying critical portions of the environmental niche for these interactions to take place. The results in the environmental niche where explicitly translated to its spatial counterpart for examining territories where the presence of the tick and the circulation of the pathogens could be restricted by an insufficient availability of vertebrates.

## Materials and methods

### Background

The study addresses the predicted interactions between the tick *I. ricinus* and a set of 162 species of vertebrates, which are either hosts for the tick or reservoirs for *B. burgdorferi* s.l., which is transmitted by the tick. The aim is to establish the portions of the niche were interactions are high and foci persist. All the data were previously compiled, reported, and assessed for reliability (Estrada-Peña and de la Fuente, 2016). The complete set of data has been already published (i.e., Estrada-Peña et al., 2013; Estrada-Peña and de la Fuente, 2016) further updated for this study, and is available in a public repository (http://datadryad.org/resource/doi:10.5061/dryad.2h3f2). In this study we aimed to (i) capture the suitability of the environment for the tick and each species of the vertebrate in the Western Palaearctic, (ii) compute an index of habitat overlap of the tick with each species of vertebrate, (iii) weight the index before according to the importance of each species of vertebrate for either the tick or the pathogen, (iv) project the results in the environmental niche, and (v) to project the results in the spatial (geographical) niche. The results must to be interpreted as the predicted circulation of the pathogen in either the dimensions of the niche or its spatial translation.

### Set of distribution data of *I. ricinus* and its hosts

Data on *I. ricinus* were compiled from literature references published since the year 1990. Data reporting either questing ticks or feeding on hosts were included. In the first case, only data including reliable coordinates or an unambiguous locality name (which was later resolved to coordinates) were considered. In the second, only data mentioning the species of hosts were further processed if adequately geo-referenced.

We downloaded the data of the recorded distribution of the vertebrates reported as hosts or reservoirs from the Global Biodiversity Information Facility (GBIF: http://gbif.org). The purpose of the compilation is to obtain the largest available source of recorded distributions of every vertebrate involved in the circulation of the pathogens.

### Choice of environmental variables and environmental modeling

A number of reports have argued for the use of predictors that are ecologically relevant to the target species (Glass et al., 1995; Guerra et al., 2002). In this sense, Araújo and Guisan (2006) stated that the “use of automated solutions to predictor selection…should not be seen as a substitution for preselecting sound eco-physiological predictors based on deep knowledge of the bio-geographical and ecological theory.” We already expressed our concerns about the reliability of interpolated variables in the building of predictive models (Estrada-Peña et al., 2016) and satellite-derived information seems to be far more robust than interpolated measures of climate, which otherwise retain its value to explain the weather conditions in a given interval of time. We adhered to published protocols (Estrada-Peña and de la Fuente, 2016) to obtain a time series of MODIS-derived satellite data regarding land surface temperature (LSTD) and the Normalized Difference Vegetation Index (NDVI) which is a measure of the photosynthetic activity of the vegetation. It has been reported that variables derived from these two basic measurements of the environment are able to capture a large fraction of the factors driving the distribution of organisms (Estrada-Peña et al., 2015a). We used data at 16-days intervals spanning the period 2001–2015, which were subjected to a Fourier transformation (or harmonic regression). After the transformation, the average, maximum and minimum LSTD and NDVI were used for modeling purposes.

Probabilities of occurrence of both the tick and the hosts were produced using correlative modeling. We calculated the expected environmental suitability for the tick and the hosts using the pairs of coordinates for the recorded distribution of every organisms (as reported by Estrada-Peña and de la Fuente, 2016; and available in http://datadryad.org/resource/doi:10.5061/dryad.2h3f2). We independently modeled the distribution of each species using the niche modeling algorithm MaxEnt integrated in the package dismo for R (Hijmans et al., 2017). This modeling algorithm demonstrated robust performance when presence-only data is available. Models were developed with lineal and quadratic features, with a maximum of 10,000 background points, 10 replicates per species modeled, and 70% of points for training purposes, using cross-validation to compare the resulting models. The regularization multiplier was set to 1. Each model was replicated 100 times using the cross-validation function in MaxEnt to partition the data into replicate folds.

### Modeling of the biotic interactions between the tick and hosts

The procedure above produces an estimation of the probable distribution of each organism. However, biotic interactions between the tick and the hosts are not taken into account. It is therefore necessary not only to evaluate the amount of habitat overlap between the tick and each host, but also to weight that ratio by the estimated interaction between the partners. It has been reported that the networks theory can provide light to evaluate these relationships (Estrada-Peña et al., 2015b). The basic tenets of network theory evaluate interactions between “nodes” (i.e., organisms) using the number of times such relationship has been recorded. It has been proposed (Estrada-Peña and de la Fuente, 2016) that a Centrality-Weighted habitat overlap (CWho) index is a simple definition of (i) the habitat overlap between the tick and each species of hosts, and (ii) the number of recorded interactions that weights the crude value of habitat overlapping. The readers are referred to the previous publication for the complete derivation and rationale of the index.

Results from the CWho must to be interpreted as “expected probabilities of interactions” between the tick, and the host(s). These interactions are derived from the habitat overlap between tick and host(s) weighted by the relative importance of each vertebrate as host(s) for the tick or as reservoir(s) for the pathogen. Thus, high values of the CWho mean for high predicted interactions resulting in a large circulation of the pathogen. Low values of CWho may result from a low habitat overlap of the tick and host(s) or from the low importance of a vertebrate for either the tick or the pathogen. The methodology introduced allows the expression of these values in either the environmental or the spatial dimensions. This immediately provides an estimation of these predicted interactions in the climate or spatial gradients.

### Spatial processing and plotting on the environmental niche

**Figure 1:**
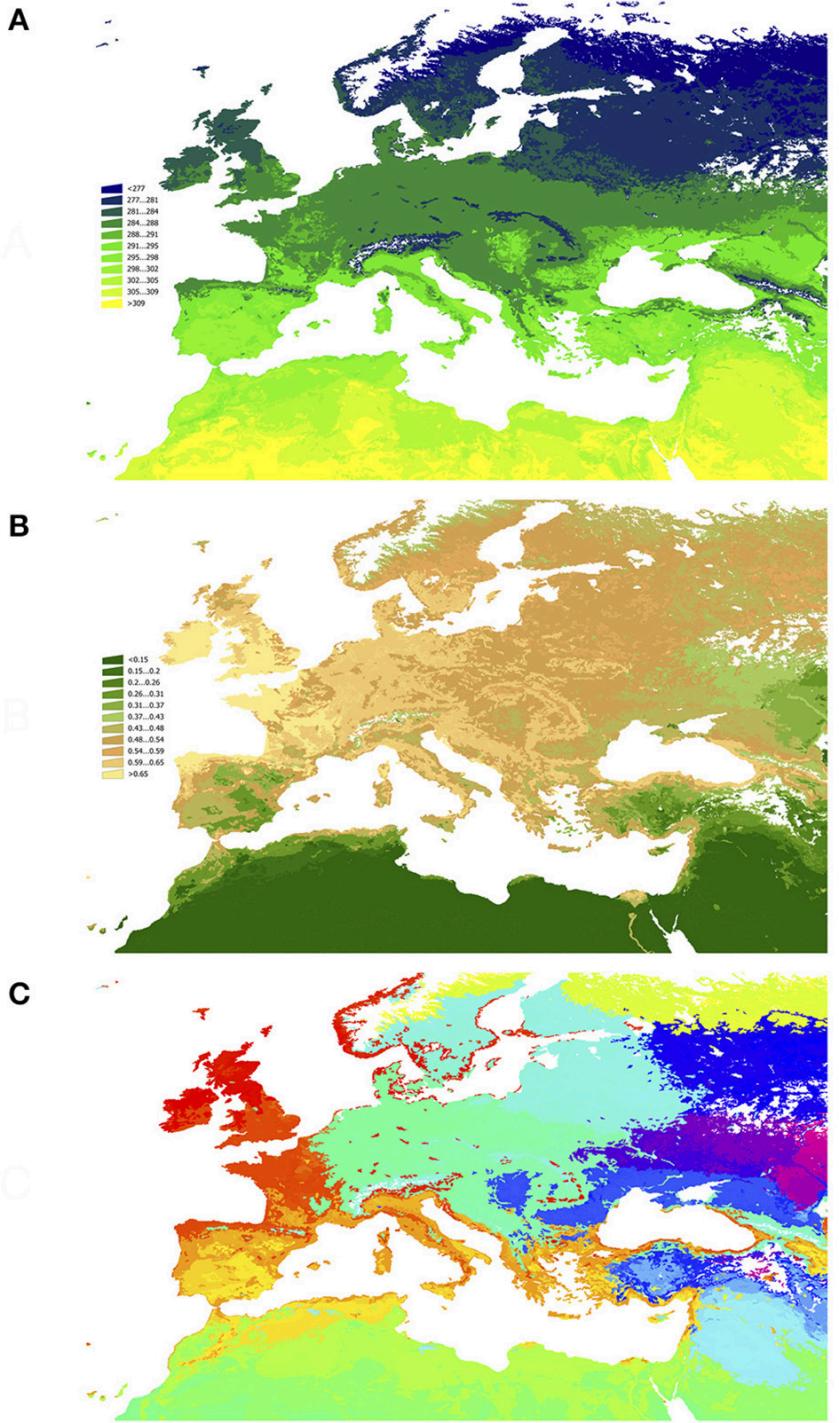
A map of the target territory with the gradient of land surface temperature (**A**, in Kelvin), and of NDVI (**B**, unit less), and **(C)** the categories of the territory (sites) according to the gradients in **(A,B)**, after disaggregation using a Principal Components Analysis (sites colored consecutively from blue to yellow).

The environmental niche is a gradient of *n* dimensions that equal the number of variables used for its definition. To obtain a tractable framework without losing reliability it is necessary to divide the gradient of the niche into categories. We used two multifactorial methods to (i) reduce the number of dimensions and divide the territory in “categories of niche” using a Principal Components Analysis (PCA) approach, and (ii) associate the results of the predictive modeling of each species of hosts with the environmental using a Canonical Correspondence Analysis (CCA). The first method produces “categories of niche” and the second associates the CWho to these categories. In this application, PCA takes the 6-dimensions environmental niche and produces a number of categories using recursive rules. Each category is thus composed by the portions of the niche gradient that are more similar between them than with the other, always considering the 6 dimensions. Figure 1 shows the spatial distribution of the average LSTD and NDVI in the target territory, and how the combination of the six variables reduced by PCA aggregate into sites.

The use of the sites emanating from a PCA has the immediate application of associating the interactions between organisms with the different sites in a simple two-axes chart. This results in the mapping of the CWho along the reduced environmental niche. We choose CCA as the ordination technique to derive biologically scaled responses along environmental gradients: this results in the association between “sites” (which represent the zonation obtained from the environmental gradients) and the “organisms” (which are the hosts of *I. ricinus* or reservoirs for *B. burgdorferi s.l*.). Since its original development (ter Braak, 1986) CCA has been already used to describe a variety of ecological communities, together with the factors that shape them, and to display the relationships between survey points and environmental variables prevailing at the collections points (Dumbrell et al., 2010; Legendre et al., 2011). The method has been adequately tested under several conditions and proved to be enough robust and confident (Gittins, 2012; ter Braak, 2014).

## Results

### The categories of climate and the environmental suitability for hosts

**Figure 2:**
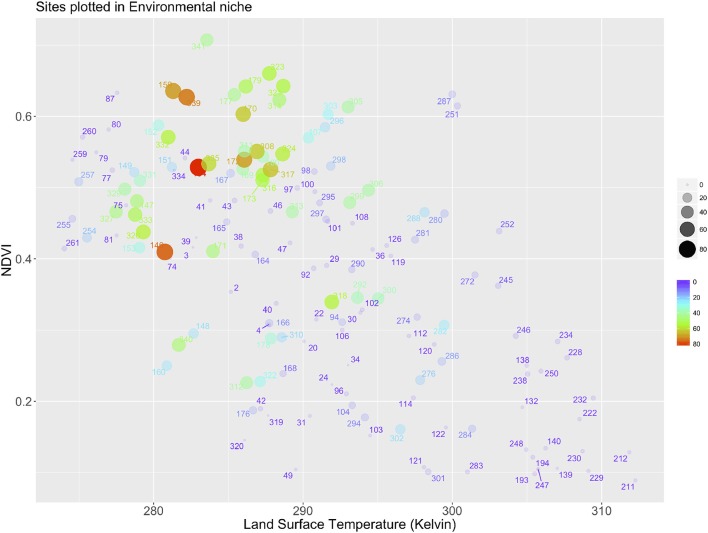
The plot of the sites in Western Palaearctic, along the two axes delimiting land surface temperature and the NDVI. Each dot corresponds to one of the sites produced by aggregation of the gradients of the environmental variables after a Principal Components Analysis. Each dot represents the position of one site according to its values of average of LST and NDVI. The color, the size and the transparency (to improve readability) of each dot is proportional to the Centrality-Weighted habitat overlap of the complete set of hosts.

The aggregation of the territory along the environmental gradients of LSTD and NDVI produced a total of 340 sites (Figures 1A–C). Each correlatively colored region in Figure 1C represents a portion of the territory that has a statistically significant different combination of both LSTD and NDVI. Figure 2 shows the distribution of all the sites in the environmental space of the average LSTD × NDVI, with the values of CWho. The plot shows each site correlatively numbered: the position of each site corresponds to its values of LSTD (X axis) and NDVI (Y axis). The size, color and transparency of each site correspond to the predicted interactions, collectively for all vertebrates. Relatively high values of CWho exist in large regions of the environmental niche. A cluster of sites with the highest CWho appears at colder (280–290 K) and wet (NDVI = 0.4–0.7) portions of the niche, with isolated spots of high CWho in the warmer and drier portions of the niche. However, CWho clearly decreases in the warmest and driest part of the environmental gradient.

### The interactions in the reduced environmental space

**Figure 3:**
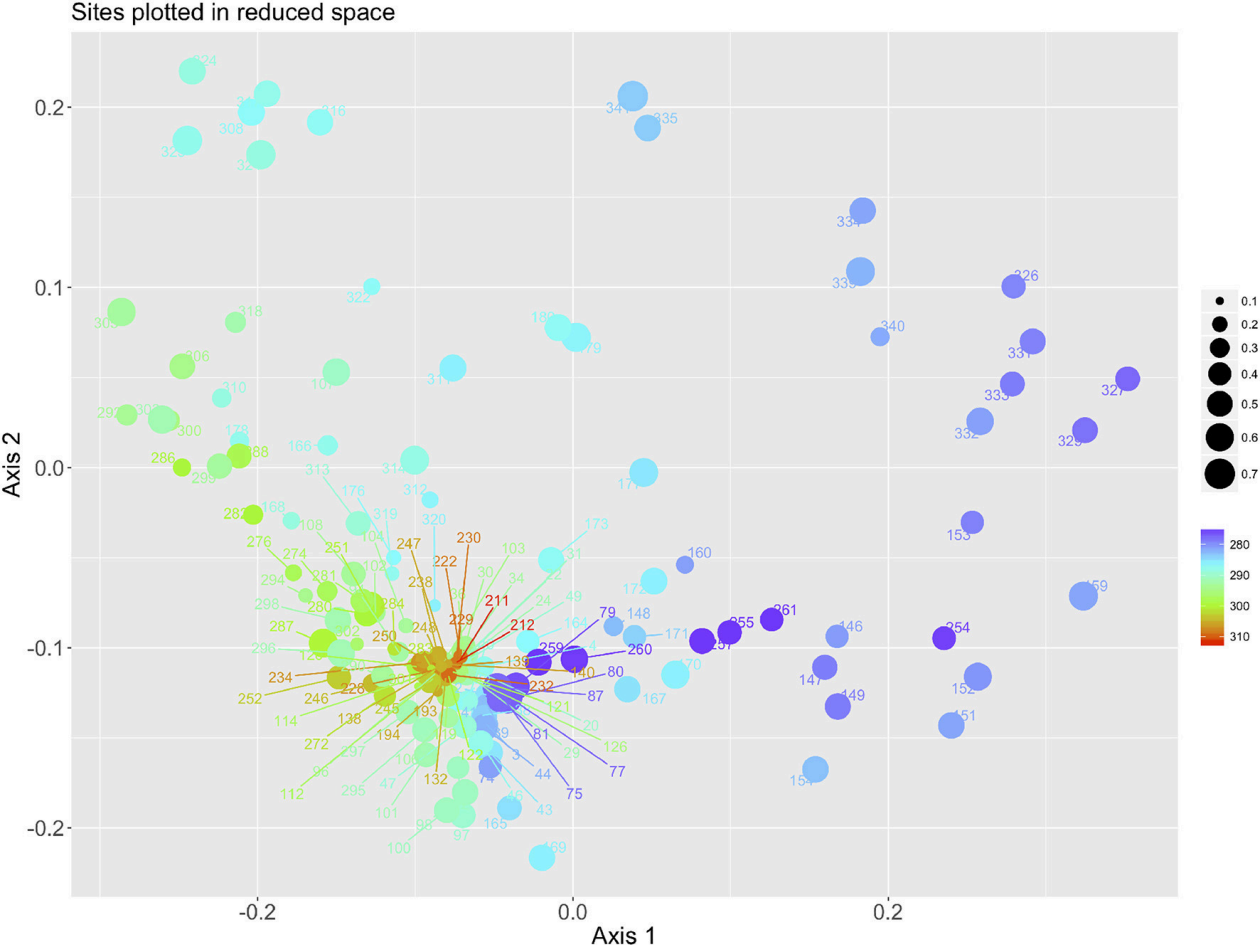
The plot of the sites of Western Palaearctic, along the two axes obtained after a Canonical Correspondence Analysis of the 6 variables of LST and NDVI. Each dot corresponds to one of the sites produced by aggregation in categories along the gradients of the environmental variables and represents the position of one site in the reduced niche. The color is proportional to the temperature range (in Kelvin), the size is proportional to the NDVI (unit less).

**Figure 4:**
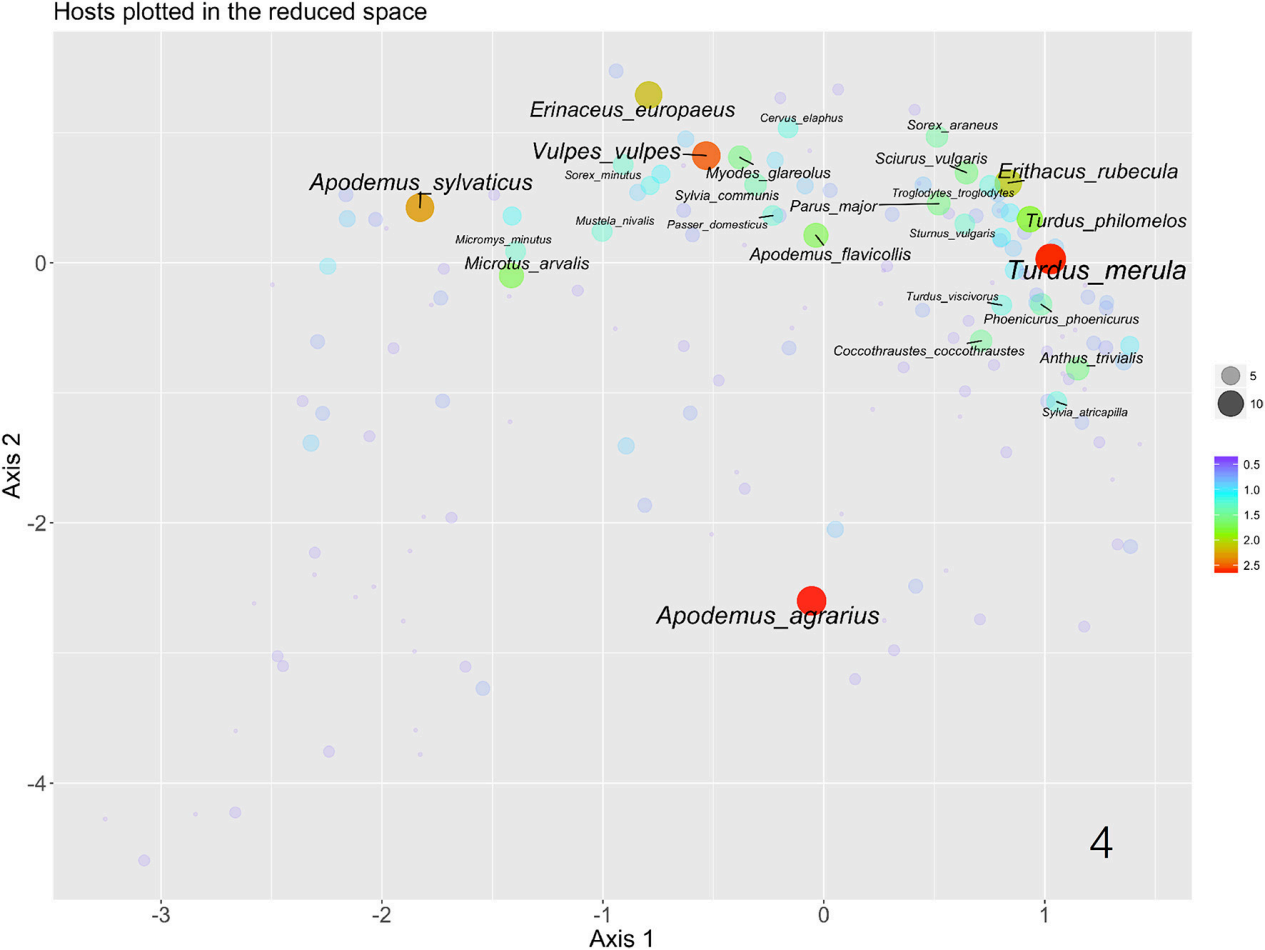
The plot of the species of vertebrates involved in the support of *I. ricinus* and *B. burgdorferi* s.l. in Western Palaearctic, along the two axes obtained after a Canonical Correspondence Analysis of the 6 variables of LST and NDVI. Each dot corresponds to a species of vertebrate and represents its position in the reduced niche (to be compared with the values of LST and NDVI as shown in the previous chart). The color and the size are proportional to the Centrality-Weighted habitat overlap. Only the top 23 species of vertebrates in the range of importance for the circulation of *B. burgdorferi* s.l. are labeled.

Figure 3 plots the sites according to the reduction of the 6 environmental variables after a PCA, using the color and the size of the symbols to display the average LSTD and NDVI of each site, respectively. The axis X is mainly driven by the temperature, with colder sites at the right of the ordination and warmer sites at the left. The values of NDVI have smaller importance in this ordination, and variability follows mainly the axis Y. This plot of sites associates immediately with the plot of the CWho, as displayed in the Figure 4. To improve the readability of the chart we labeled only the 23 most prominent species of vertebrates for the circulation of *B. burgdorferi* s.l. A clear patterns appears: interactions with most prominent reservoirs of *B. burgdorferi* are associated with a variable range of temperature (appearing along most of the range of the X axis) but in the portions of the chart corresponding with medium and high values of NDVI. *Apodemus agrarius* is however separated of that gradient and interactions associate with cold portions of the niche and medium values of NDVI. The remaining 133 species of hosts are restricted to portions of higher LSTD and lower values of NDVI (Figure 4).

### The interactions in the geographic space

**Figure 5:**
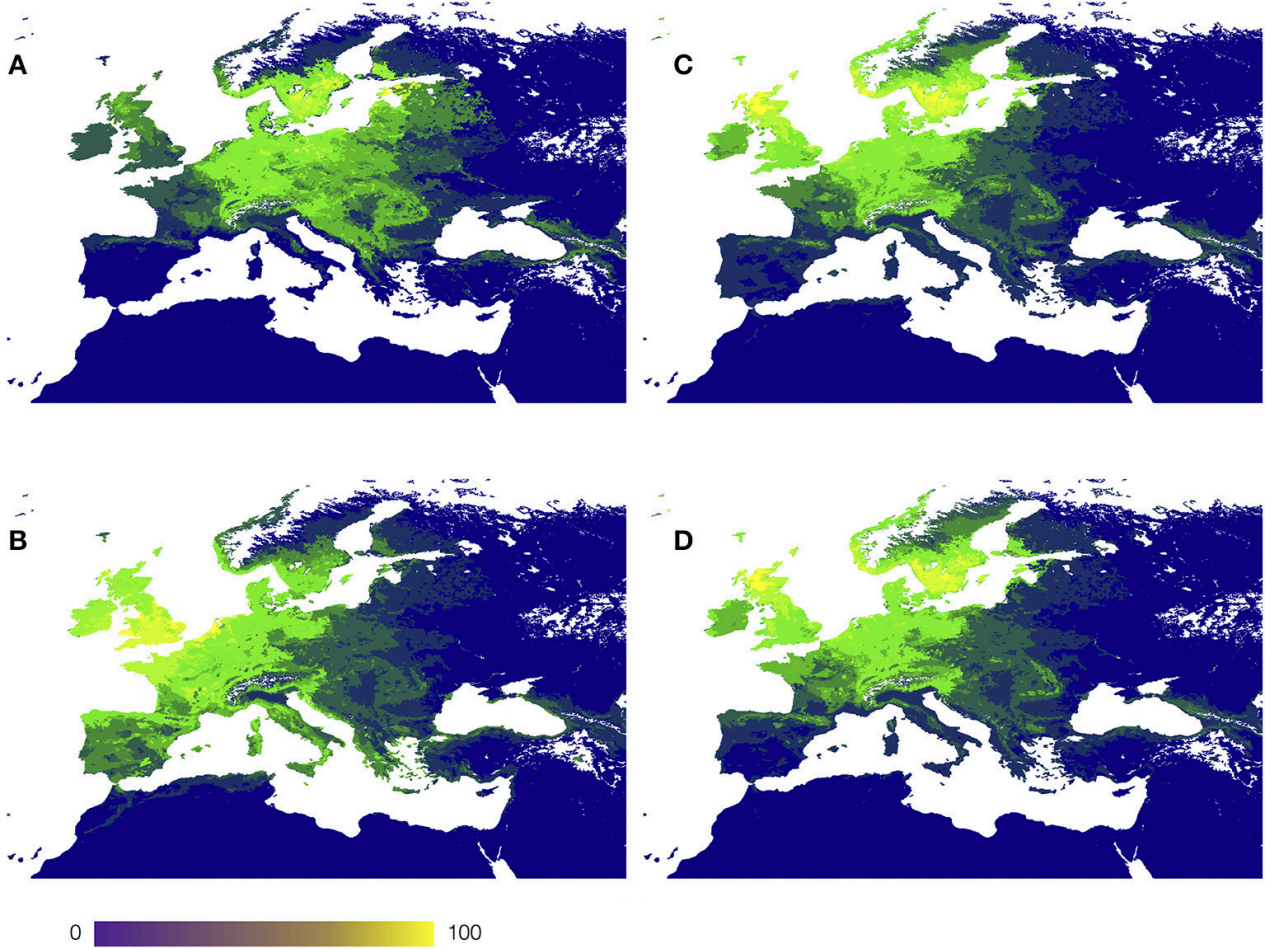
The plot of the Centrality-Weighted habitat overlap for *B. burgdorferi* s.l. in Western Palaearctic with four selected species of vertebrates, rescaled to the range 0–100 for comparability. **(A)** *Apodemus agrarius*; **(B)** *Apodemus sylvaticus*; **(C)** *Turdus merula*; **(D)** *Turdus philomelos*.

**Figure 6:**
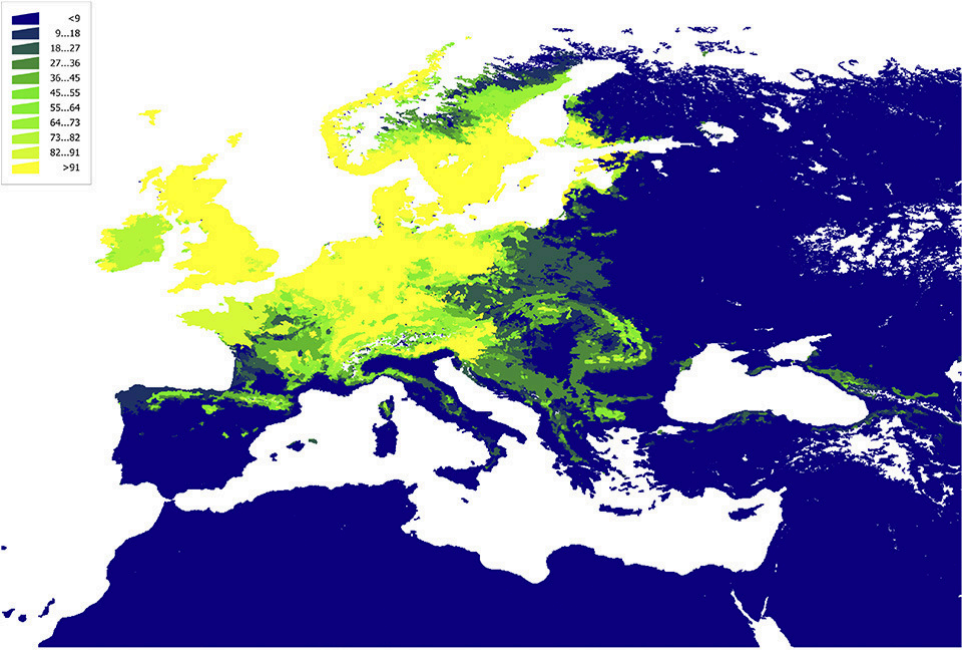
The plot of the Centrality-Weighted habitat overlap for *B. burgdorferi* s.l. in Western Palaearctic with Aves, Passeriformes, rescaled to the range 0–100 for comparability.

**Figure 7:**
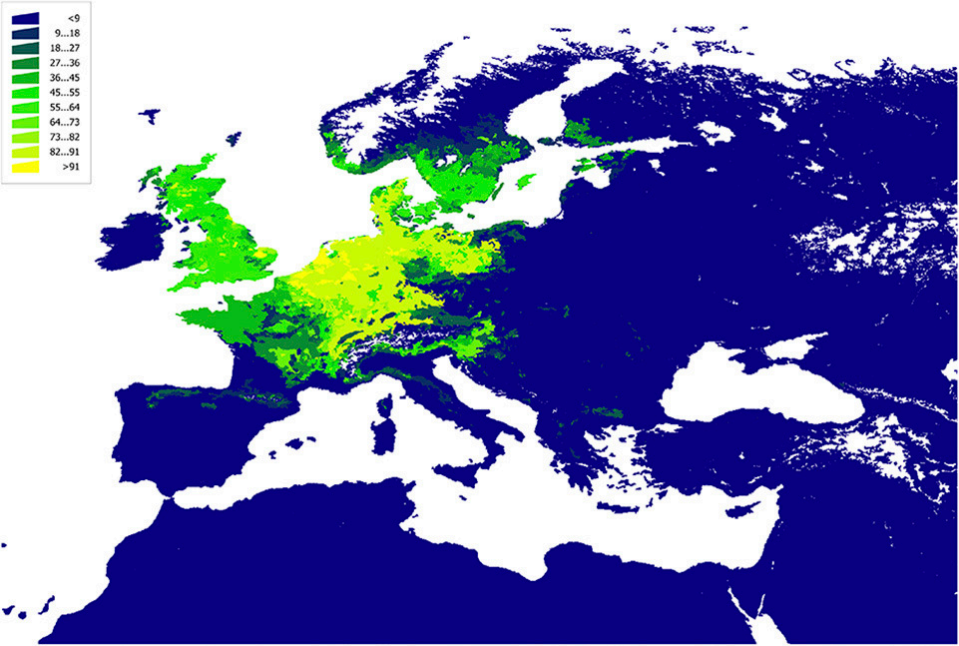
The plot of the Centrality-Weighted habitat overlap for *B. burgdorferi* s.l. in Western Palaearctic for Rodentia, rescaled to the range 0–100 for comparability.

The CWho was translated from the environmental gradient into the geographic space to capture the spatial gradient of variability in the interactions. These results were summarized in Figures 5–7, including the expected interactions with two species of Rodentia: Muridae (*Apodemus sylvaticus* and *A. flavicollis*) and two species of Passeriformes: Turdidae (*Turdus merula* and *T. phylomelos*) (Figures 5A–D), the expected interactions with Aves: Passeriformes (Figure 6), and the same values for Mammalia: Rodentia (Figure 7). The results clearly indicated that large portions of the territory are suitable resulting in a potential circulation of the pathogen in a wide territory.

## Discussion

This study showed that *B. burgdorferi* s.l. persists in the Western Palaearctic because of a redundancy of the interactions vertebrates that are both suitable hosts for the tick vector and adequate reservoirs of the pathogen. Even if the top reservoirs are absent in a territory, the wide availability of vertebrates considered secondary reservoirs contribute to the persistence of the pathogen. The role of biotic interactions has been classically discussed as one of the factors driving the distribution of ticks but its importance for the circulation of pathogens has been neglected. We specifically focused this study on *I. ricinus* and *B. burgdorferi* s.l. because its impact on human health (i.e., Medlock et al., 2013; Jahfari et al., 2014; Biernat et al., 2016; Radzijevskaja et al., 2016). *Ixodes ricinus* is one of the species of ticks that transmits *B. burgdorferi* s.l. and feeds on a wide spectrum of hosts, with a variable feeding success because their innate resistance to the tick (Pérez et al., 2016; van Duijvendijk et al., 2016; Van Oosten et al., 2016). These tick-vertebrate contact rates are primarily governed by the prevailing climate, which shapes the abundance of the hosts and the tick (Estrada-Peña et al., 2014) further modulated by a complex set of factors including the immune system plus morphological and physiological traits of the vertebrates (Barbour et al., 2015; Hofmeester et al., 2016). Many studies have demonstrated that the relative faunal composition of vertebrates is a key factor driving the community of this pathogen (Rudenko et al., 2014) and recent reports addressed meta-analyses of the relative importance of the reservoirs in the maintenance of active foci (i.e., Barbour et al., 2015; van Duijvendijk et al., 2015; Kikpatrick et al., 2017). However, a predictive approach for modeling Lyme borreliosis utilizing *separately* the abiotic interactions of the tick and the environment, the contact rates between ticks and vertebrates, and the reservoir capacity of the vertebrates, is unreliable (Estrada-Peña and de la Fuente, 2016). A holistic approach is necessary for understanding the patterns of circulation of the pathogen (Franke et al., 2013; Schotthoefer and Frost, 2015).

We formulated a theoretical background derived from a large dataset of interactions between ticks, pathogens and vertebrate reservoirs, demonstrating that ecological relationships between partners could be mapped in the environmental niche to extract relevant epidemiological information (Estrada-Peña and de la Fuente, 2016). The method has a strict mathematical foundation and can evaluate the relative importance of each vertebrate species supporting the circulation of a tick-transmitted pathogen. We herein demonstrated that (i) the plotting on the reduced space is suitable for understanding the relationships between vertebrates and their niches, (ii) the use of a CCA provides a coherent framework to demonstrate these links, (iii) *I. ricinus* does not depend on a few hosts to colonize different portions of the environmental niche, since literally every portion of that niche is filled with a large array of hosts, and (iv) prevailing weather shapes the patterns of circulation of the complex *B. burgdorferi* s.l. supporting a large redundancy of the vertebrates allowing permanent foci. This conclusion is of special interest since local or regional analyses of the reservoir capacity of the vertebrates could not be extrapolated to other regions where the pathogen circulates. Our study has explicitly restricted the analyses to the environmental niche, with only a partial translation of the results to the spatial (geographical) domain as a proof of concept. The extrapolation of results to the geographical dimensions is of special complexity giving the genetic diversity and competition events among the reservoirs established at each site, which has a direct impact on the circulation of the pathogen (Becker et al., 2016). This results in a complex circumstance in which risk assessment in the space is necessary for human health managers, but for which our level of knowledge is far from complete.

The results showed that the wide circulation of *B. burgdorferi* s.l. in Western Palaearctic arises from a large number of vertebrate species covering literally every portion of the niche available for the tick vector. Everywhere *I. ricinus* exists, a considerable variety of vertebrates exist, amplifying the transmission of the pathogen. Therefore, the circulation of *B. burgdorferi* s.l. is based on the concept of functional redundancy, something that escaped to meta-analyses. Since the niche produces an obvious gradient of suitability (and therefore abundance) of the main reservoirs, variable prevalence of infected ticks with different species of *Borrelia* should be expected. This fact produces the already reported regional differences in the prevalent *Borrelia* spp. (van Duijvendijk et al., 2015; Pérez et al., 2016; Ruyts et al., 2016). It is necessary to stress that the circulation of *B. burgdorferi* s.l. has been never tested in the context of the complete community of vertebrates, most extensive data coming from areas in the United States (i.e., LoGiudice et al., 2003). This is of significance because in the absence of a vertebrate, other(s) could assume a different role as hosts for the tick, which may have different capacities for supporting the circulation of the pathogen. Available reports and meta-analyses refer commonly to the most easily trapped subset of vertebrates, since some others may be especially difficult to trap or constitute endangered species. These procedures would under-represent some vertebrates, potentially resulting in a distortion of the perception of the tick-vertebrate-pathogen system. This “context view” would be desirable to further contribute to the understanding of any tick-pathogen system (Hofmeester et al., 2016).

The results of this study stressed the descriptive abilities of a network of interactions between ticks and vertebrates for supporting foci of a tick-borne pathogen on large portions of the abiotic niche. This conclusion challenges the classic dogma of only a few vertebrates supporting the stability of the circulation of *B. burgodrferi* s.l. (States et al., 2014; Hofmeester et al., 2016). Our results demonstrated that the generalist feeding habits of the tick and the large availability of the reservoirs provide the substratum where the pathogen largely circulates. Environmental forces that affect niche overlap between the tick and the vertebrates shape the gradient of transmission; it is unrealistic to observe these transmission forces under the scope of a reduced set of reservoirs. Therefore, one of our main conclusion is that reservoir competence analyses obtained from regional studies, which are invaluable to understand local circulation rates of the pathogen, are meaningless if extrapolated to different regions. Most of the niche where the tick survives is filled with a high diversity of vertebrates, allowing the circulation of the pathogen at variable rates according to the genetic composition of the prevailing fauna. Their contributions, together, largely support the wide circulation of the pathogen in the target territory. We explicitly recommend the exploration of the environmental niche to characterize the circumstance under which a tick-borne pathogen can be prevalent, before exploring the spatial relationships of its distribution.

This framework could be applied to other species of ticks, or, perhaps of most importance, to integrate the current knowledge of the ecological relationships between the species of ticks transmitting the different genospecies of *B. burgdorferi* s.l. in the world. This would provide an overview of the large amount of data so far reported from regional surveys. Quantitative data about reservoir abilities of each vertebrate as well as explicit considerations of their abilities to support the feeding of ticks should be included in further improvements of this framework.

## Author contributions

Both AE-P and JdlF designed the experiments, contributed to the development of the study, and wrote the paper.

### Conflict of interest statement

The authors declare that the research was conducted in the absence of any commercial or financial relationships that could be construed as a potential conflict of interest.
